# Key transmission parameters of an institutional outbreak during the 1918 influenza pandemic estimated by mathematical modelling

**DOI:** 10.1186/1742-4682-3-38

**Published:** 2006-11-30

**Authors:** Gabriel Sertsou, Nick Wilson, Michael Baker, Peter Nelson, Mick G Roberts

**Affiliations:** 1 Department of Public Health, Wellington School of Medicine & Health Sciences, University of Otago, Wellington, New Zealand; 2 Centre for Mathematical Biology, Institute of Information and Mathematical Sciences, Massey University, Auckland, New Zealand

## Abstract

**Aim:**

To estimate the key transmission parameters associated with an outbreak of pandemic influenza in an institutional setting (New Zealand 1918).

**Methods:**

Historical morbidity and mortality data were obtained from the report of the medical officer for a large military camp. A susceptible-exposed-infectious-recovered epidemiological model was solved numerically to find a range of best-fit estimates for key epidemic parameters and an incidence curve. Mortality data were subsequently modelled by performing a convolution of incidence distribution with a best-fit incidence-mortality lag distribution.

**Results:**

Basic reproduction number (*R*_0_) values for three possible scenarios ranged between 1.3, and 3.1, and corresponding average latent period and infectious period estimates ranged between 0.7 and 1.3 days, and 0.2 and 0.3 days respectively. The mean and median best-estimate incidence-mortality lag periods were 6.9 and 6.6 days respectively. This delay is consistent with secondary bacterial pneumonia being a relatively important cause of death in this predominantly young male population.

**Conclusion:**

These *R*_0 _estimates are broadly consistent with others made for the 1918 influenza pandemic and are not particularly large relative to some other infectious diseases. This finding suggests that if a novel influenza strain of similar virulence emerged then it could potentially be controlled through the prompt use of major public health measures.

## Background

The 1918 influenza pandemic reached New Zealand with an initial wave between July and October [1]. This was relatively mild with only four deaths out of 3048 reported cases for the population of military camps [1]. The second wave in late October was much more severe and spread throughout the country causing over 8000 deaths [2]. One large military camp near Featherston (a town in the south of the North Island) also suffered from exposure to the second wave of the 1918 pandemic at approximately the same time as the rest of the country. Influenza cases were reported in the camp from 28 October to 22 November 1918, and reported mortality occurred between 7 November and 11 December 1918, with both incidence and mortality peaking in November 1918 [2]. A unique feature of this military camp outbreak was the systematic collection by medical staff of morbidity data as well as mortality data. We undertook modelling of these data to understand better the transmission dynamics of the 1918 influenza pandemic in New Zealand.

## Methods

### Data

The population of the Featherston Military Camp was that of a large regional town, comprising approximately 8000 military personnel of whom 3220 were hospitalised [3]. The camp policy was to hospitalise all those with diagnosed influenza and so we have used these hospitalisation data as the basis for the incidence of pandemic influenza in this population. An official report indicated a total of 177 deaths attributable to the outbreak [4]. However, this figure was actually the total number of men who died in the camp in 1918 from all causes as reported by the Principal Medical Officer at the camp [3]. Further examination of data on the cause of death and date-of-death suggests the total mortality attributable to this outbreak was 163 [5]. This revision gives a fairly conservative figure for the mortality impact and it is the one that we have used in this analysis.

### Mathematical modelling approach

A susceptible-exposed-infectious-recovered (SEIR) model for infectious diseases can be applied to a hypothetical isolated population, to investigate local infection dynamics [6,7]. The SEIR model allows a systematic method by which to quantify the dynamics, and derive epidemiological parameters for disease outbreaks. In this model, individuals in a hypothetical population are categorized at any moment in time according to infection status, as one of susceptible, exposed, infectious, or removed from the epidemic process (either recovered and immune or deceased). If an infected individual is introduced into the population, rates of change of the proportion of the population in each group (*s*, *e*, *i*, and *r*, respectively) can be described by four simultaneous differential equations:

dsdt=−βsi     (1)
 MathType@MTEF@5@5@+=feaafiart1ev1aaatCvAUfKttLearuWrP9MDH5MBPbIqV92AaeXatLxBI9gBaebbnrfifHhDYfgasaacH8akY=wiFfYdH8Gipec8Eeeu0xXdbba9frFj0=OqFfea0dXdd9vqai=hGuQ8kuc9pgc9s8qqaq=dirpe0xb9q8qiLsFr0=vr0=vr0dc8meaabaqaciaacaGaaeqabaqabeGadaaakeaadaWcaaqaaiabdsgaKjabdohaZbqaaiabdsgaKjabdsha0baacqGH9aqpcqGHsisliiGacqWFYoGycqWGZbWCcqWGPbqAcaWLjaGaaCzcamaabmaabaGaeGymaedacaGLOaGaayzkaaaaaa@3C60@

dedt=βsi−νe     (2)
 MathType@MTEF@5@5@+=feaafiart1ev1aaatCvAUfKttLearuWrP9MDH5MBPbIqV92AaeXatLxBI9gBaebbnrfifHhDYfgasaacH8akY=wiFfYdH8Gipec8Eeeu0xXdbba9frFj0=OqFfea0dXdd9vqai=hGuQ8kuc9pgc9s8qqaq=dirpe0xb9q8qiLsFr0=vr0=vr0dc8meaabaqaciaacaGaaeqabaqabeGadaaakeaadaWcaaqaaiabdsgaKjabdwgaLbqaaiabdsgaKjabdsha0baacqGH9aqpiiGacqWFYoGycqWGZbWCcqWGPbqAcqGHsislcqWF9oGBcqWGLbqzcaWLjaGaaCzcamaabmaabaGaeGOmaidacaGLOaGaayzkaaaaaa@3F4C@

didt=νe−γi     (3)
 MathType@MTEF@5@5@+=feaafiart1ev1aaatCvAUfKttLearuWrP9MDH5MBPbIqV92AaeXatLxBI9gBaebbnrfifHhDYfgasaacH8akY=wiFfYdH8Gipec8Eeeu0xXdbba9frFj0=OqFfea0dXdd9vqai=hGuQ8kuc9pgc9s8qqaq=dirpe0xb9q8qiLsFr0=vr0=vr0dc8meaabaqaciaacaGaaeqabaqabeGadaaakeaadaWcaaqaaiabdsgaKjabdMgaPbqaaiabdsgaKjabdsha0baacqGH9aqpiiGacqWF9oGBcqWGLbqzcqGHsislcqWFZoWzcqWGPbqAcaWLjaGaaCzcamaabmaabaGaeG4mamdacaGLOaGaayzkaaaaaa@3DED@

drdt=γi     (4)
 MathType@MTEF@5@5@+=feaafiart1ev1aaatCvAUfKttLearuWrP9MDH5MBPbIqV92AaeXatLxBI9gBaebbnrfifHhDYfgasaacH8akY=wiFfYdH8Gipec8Eeeu0xXdbba9frFj0=OqFfea0dXdd9vqai=hGuQ8kuc9pgc9s8qqaq=dirpe0xb9q8qiLsFr0=vr0=vr0dc8meaabaqaciaacaGaaeqabaqabeGadaaakeaadaWcaaqaaiabdsgaKjabdkhaYbqaaiabdsgaKjabdsha0baacqGH9aqpiiGacqWFZoWzcqWGPbqAcaWLjaGaaCzcamaabmaabaGaeGinaqdacaGLOaGaayzkaaaaaa@3A0E@

where *β*, *ν *and *γ *are rate constants for transformation of individuals from susceptible to exposed, from exposed to infectious, and from infectious to recovered and immune states, respectively. Once the above equations have been solved, the parameters *β *and *γ *can be utilized to calculate the basic reproduction number (*R*_0_) for the particular virus strain causing the outbreak. (The basic reproduction number represents the number of secondary cases generated by a primary case in a completely susceptible population). *R*_0 _and the average latent period (*T*_*E*_), and average infectious period (*T*_*I*_), can be calculated using the following relationships:

R0=βγ     (5)
 MathType@MTEF@5@5@+=feaafiart1ev1aaatCvAUfKttLearuWrP9MDH5MBPbIqV92AaeXatLxBI9gBaebbnrfifHhDYfgasaacH8akY=wiFfYdH8Gipec8Eeeu0xXdbba9frFj0=OqFfea0dXdd9vqai=hGuQ8kuc9pgc9s8qqaq=dirpe0xb9q8qiLsFr0=vr0=vr0dc8meaabaqaciaacaGaaeqabaqabeGadaaakeaacqWGsbGudaWgaaWcbaGaeGimaadabeaakiabg2da9maalaaabaacciGae8NSdigabaGae83SdCgaaiaaxMaacaWLjaWaaeWaaeaacqaI1aqnaiaawIcacaGLPaaaaaa@3722@

TE=1ν     (6)
 MathType@MTEF@5@5@+=feaafiart1ev1aaatCvAUfKttLearuWrP9MDH5MBPbIqV92AaeXatLxBI9gBaebbnrfifHhDYfgasaacH8akY=wiFfYdH8Gipec8Eeeu0xXdbba9frFj0=OqFfea0dXdd9vqai=hGuQ8kuc9pgc9s8qqaq=dirpe0xb9q8qiLsFr0=vr0=vr0dc8meaabaqaciaacaGaaeqabaqabeGadaaakeaacqWGubavdaWgaaWcbaGaemyraueabeaakiabg2da9maalaaabaGaeGymaedabaacciGae8xVd4gaaiaaxMaacaWLjaWaaeWaaeaacqaI2aGnaiaawIcacaGLPaaaaaa@36B2@

TI=1γ     (7)
 MathType@MTEF@5@5@+=feaafiart1ev1aaatCvAUfKttLearuWrP9MDH5MBPbIqV92AaeXatLxBI9gBaebbnrfifHhDYfgasaacH8akY=wiFfYdH8Gipec8Eeeu0xXdbba9frFj0=OqFfea0dXdd9vqai=hGuQ8kuc9pgc9s8qqaq=dirpe0xb9q8qiLsFr0=vr0=vr0dc8meaabaqaciaacaGaaeqabaqabeGadaaakeaacqWGubavdaWgaaWcbaGaemysaKeabeaakiabg2da9maalaaabaGaeGymaedabaacciGae83SdCgaaiaaxMaacaWLjaWaaeWaaeaacqaI3aWnaiaawIcacaGLPaaaaaa@36AB@

Other factors that are likely to affect the observed incidence of disease in a pandemic include the following: (i) the initial proportion of population that is susceptible (*P*_*is*_); (ii) the proportion of infected cases who develop symptoms (*P*_*ids*_); (iii) the infectivity of asymptomatic people relative to the infectivity of symptomatic people (*Inf*_*as*_); and (iv) the proportion of symptomatic cases who present (*P*_*sp*_).

In this study, the factors listed above were incorporated into an SEIR model to generate incidence and subsequent mortality models for the influenza pandemic that swept through this military camp. These specific models and the resulting estimates of *R*_0 _and *T*_*E *_and *T*_*I *_are described below.

### SEIR model of incidence

When the SEIR model was applied in this study, assumptions about additional factors that might influence the observed incidence were made. The parameters associated with these assumptions are summarised for 3 possible scenarios (Table 1). Parameters in Scenarios 1, 2, and 3 were chosen so that models would yield estimates of *R*_0 _at the lower, mid-range and higher ends of a likely spectrum, respectively.

**Table 1:** Parameters used in the SEIR incidence model*.

**Parameter**	**Scenario 1**	**Scenario 2**	**Scenario 3**
Initial proportion of the population susceptible (*P*_*is*_)	1.0	0.9	0.8
Proportion of infected cases who develop symptoms (*P*_*ids*_)	0.95	0.81	0.67
Infectivity of asymptomatic/infectivity of symptomatic people (*Inf*_*as*_)	0.6	0.5	0.4
Proportion of symptomatic cases who present and are diagnosed as infected with influenza (*P*_*sp*_)	0.95	0.88	0.8

*Based on plausible ranges for pandemic influenza with Scenario 1 being closer to a worse case for impact on health and Scenario 3 being less severe. For example, Scenario 3 assumes 20% of the population may have had immunity from previous influenza pandemics that may have reached New Zealand in the late 19^th ^century – as suggested by Rice [2] and supported by the unusually low mortality rates in the older age groups for this pandemic in New Zealand [2].

Equations 1 and 2 were modified to take the above parameters into account, as follows:

dsdt=−β(Pids+(1−Pids)Infas)si     (8)
 MathType@MTEF@5@5@+=feaafiart1ev1aaatCvAUfKttLearuWrP9MDH5MBPbIqV92AaeXatLxBI9gBaebbnrfifHhDYfgasaacH8akY=wiFfYdH8Gipec8Eeeu0xXdbba9frFj0=OqFfea0dXdd9vqai=hGuQ8kuc9pgc9s8qqaq=dirpe0xb9q8qiLsFr0=vr0=vr0dc8meaabaqaciaacaGaaeqabaqabeGadaaakeaadaWcaaqaaiabdsgaKjabdohaZbqaaiabdsgaKjabdsha0baacqGH9aqpcqGHsisliiGacqWFYoGycqGGOaakcqWGqbaudaWgaaWcbaGaemyAaKMaemizaqMaem4CamhabeaakiabgUcaRiabcIcaOiabigdaXiabgkHiTiabdcfaqnaaBaaaleaacqWGPbqAcqWGKbazcqWGZbWCaeqaaOGaeiykaKIaemysaKKaemOBa4MaemOzay2aaSbaaSqaaiabdggaHjabdohaZbqabaGccqGGPaqkcqWGZbWCcqWGPbqAcaWLjaGaaCzcamaabmaabaGaeGioaGdacaGLOaGaayzkaaaaaa@544A@

dedt=β(Pids+(1−Pids)Infas)si−νe     (9)
 MathType@MTEF@5@5@+=feaafiart1ev1aaatCvAUfKttLearuWrP9MDH5MBPbIqV92AaeXatLxBI9gBaebbnrfifHhDYfgasaacH8akY=wiFfYdH8Gipec8Eeeu0xXdbba9frFj0=OqFfea0dXdd9vqai=hGuQ8kuc9pgc9s8qqaq=dirpe0xb9q8qiLsFr0=vr0=vr0dc8meaabaqaciaacaGaaeqabaqabeGadaaakeaadaWcaaqaaiabdsgaKjabdwgaLbqaaiabdsgaKjabdsha0baacqGH9aqpiiGacqWFYoGycqGGOaakcqWGqbaudaWgaaWcbaGaemyAaKMaemizaqMaem4CamhabeaakiabgUcaRiabcIcaOiabigdaXiabgkHiTiabdcfaqnaaBaaaleaacqWGPbqAcqWGKbazcqWGZbWCaeqaaOGaeiykaKIaemysaKKaemOBa4MaemOzay2aaSbaaSqaaiabdggaHjabdohaZbqabaGccqGGPaqkcqWGZbWCcqWGPbqAcqGHsislcqWF9oGBcqWGLbqzcaWLjaGaaCzcamaabmaabaGaeGyoaKdacaGLOaGaayzkaaaaaa@5736@

Equations 3, 4, 8 and 9 are a system of non-linear differential equations, amenable to solution by the Runge-Kutta fourth order fixed step numerical method [8]. The population size was taken to be *N *= 8000. The initial value for *s *was *P*_*is *_- 1/*N*, and initial values of *e*, *i*, and *r *were set at 0, 1/*N *and 1-*P*_*is *_respectively. The differential equation system solutions were used to calculate daily incidence, taking into account parameters in Table 1, using the following equation:

*Incidence *= *P*_*sp*_*P*_*ids*_*N*(*s*(*t *- 1) - *s*(*t*))     (10)

in which *s*(*t*) and *s*(*t-*1) are the proportion of susceptible individuals at *t *and *t-*1 days respectively after the introduction of a single symptomatic individual into the population.

For each scenario in Table 1, modelled incidence was compared to observed incidence over 26 days, and goodness of fit of the models was evaluated using sum of squared error (SSE) between modelled and empirical data. Optimum possible *β*, *ν *and *γ *values to one decimal place, in the range 0.1 to 20, were determined by finding values corresponding to a minimum SSE, utilizing an algorithm written in Mathcad [9].

The asymptotic variance-covariance matrix of the least squares estimates of *β*, *ν *and *γ*, was computed using the method described by Chowell et al. [10]. Equations 5, 6, and 7, together with elements of the variance-covariance matrix, and a Taylor series approximation for variance of quotients [11], were subsequently used to estimate best-fit values of *R*_0_, *T*_*E *_and *T*_*I*_, with associated standard deviations and confidence intervals.

### Associated mortality model

As morbidity and mortality data are not linked at the individual level, case-fatality lag was modelled by using convolution. A least-squares gamma distribution was fitted to the observed incidence curve. A gamma distribution with the same scale parameter was then fitted to mortality data. Utilising these distributions and the convolution formula, a gamma distributed incidence-mortality lag distribution, with the same scale parameter, was obtained.

Gamma distributions with the same scale parameter were then fitted to the best-fit deterministic models of daily incidence. These distributions, convolved with the incidence-mortality lag distribution, yielded daily mortality distributions for each of Scenarios 1 to 3. A common scale parameter was used in the above convolutions in order to obtain closed-form (gamma) probability density functions.

## Results

**Figure 1:**
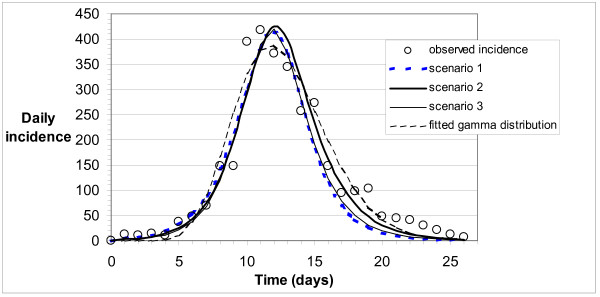
Observed and best-fit modelled incidence (ill cases per day) for Scenarios 1 to 3, and best-fit gamma distribution.

Best-fit incidence curves from the SEIR model for the three scenarios are shown in Figure 1. The corresponding best-fit *β*, *ν *and *γ*, and corresponding *R*_0_, *T*_*E *_and *T*_*I *_values, are shown in Table 2. The *R*_0 _values ranged between 1.3, and 3.1, and corresponding average latent period and infectious period estimates ranged between 0.7 and 1.3 days, and 0.2 and 0.3 days, respectively.

**Table 2:** Rate constants and epidemiological parameters corresponding to the best-fit models shown in Figure 1 (associated standard deviation or 95% confidence interval is given in brackets).

**Scenario**	***β *(days^-1^)**	***ν *(days^-1^)**	***γ *(days^-1^)**	***R*_0_**	**Latent period *T_*E *_*(days)**	**Infectious period T_*I *_(days)**
1	5.3 (0.50)	1.5 (0.08)	4.2 (0.33)	1.3 (0.02)	0.67 (0.60, 0.74)	0.24 (0.21, 0.28)
2	6.5 (0.27)	1.2 (0.04)	3.6 (0.11)	1.8 (0.04)	0.83 (0.78, 0.89)	0.28 (0.26, 0.30)
3	10.1 (1.55)	0.8 (0.11)	3.3 (0.36)	3.1 (0.18)	1.25 (0.99, 1.69)	0.30 (0.25, 0.38)

**Figure 2:**
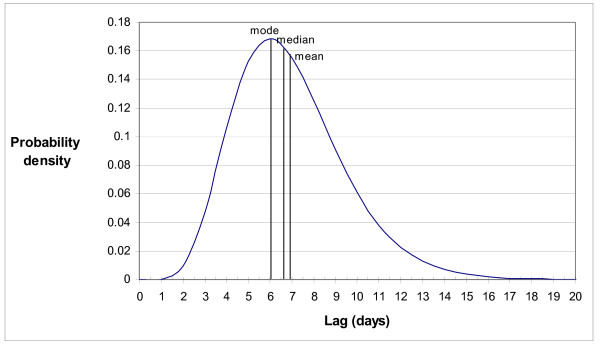
Incidence-mortality lag time distribution.

The gamma distribution of incidence-mortality lag time obtained by convolution is shown in Figure 2. The mean, median, mode and variance of this distribution are 6.9, 6.6, 6.0 and 6.3 days respectively.

**Figure 3:**
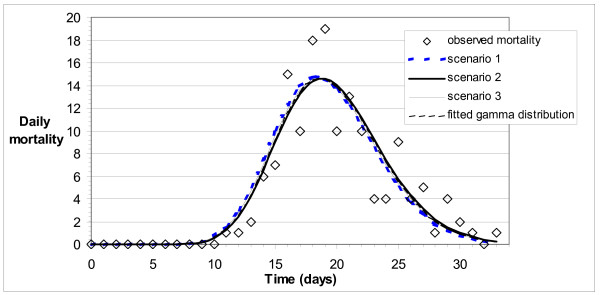
Observed and best-fit modelled mortality (deaths per day) for Scenarios 1 to 3.

Observed mortality data, shown in Figure 3, indicate more variability around a best-fit gamma distribution than observed incidence data (see Figure 1). Mortality curves for each of Scenarios 1 to 3, obtained by convolution, all agree well with the best-fit gamma distribution of observed data.

## Discussion

This analysis has demonstrated the potential for using historical disease epidemic data to derive plausible, and potentially useful, pandemic influenza parameter estimates. This is the first time that these parameters have been reported for the 1918 pandemic outside of Europe, the USA and Brazil.

### Limitations of this analysis

This work is limited by the very nature of using data from an event that occurred over eight decades ago. For example, the estimate of the camp's population was only approximate (at 8000). The mortality burden of this particular outbreak (at 20.4 per 1000) was also somewhat higher than that for the general male population of New Zealand (ie, at 10.0 per 1000 for 20–24 year olds) [2]. It was, however, similar to the pandemic influenza mortality burden of the armed forces as a whole (at 23.5 per 1000) and for other military camps at 22.0 and 23.5 (for Awapuni and Trentham camps respectively) [2]. It is plausible that higher death rates in military camps may have been related to both higher risk of infection (e.g. via crowding) and the poor living conditions involved (i.e. the extensive use of tents). Crowded troop trains may also have contributed to disease spread and in the weekend prior to the main outbreak in the camp many of the recruits had been away on leave, and were transported to and from the camp by troop trains. Furthermore, a severe storm struck the Featherston camp on 7 November (the day that influenza incidence peaked) and flattened many tents. This event placed additional stresses on accommodating men in huts that were already full and with some huts (and all institute buildings such as the YMCA, for example) being used as overflow wards to the main camp hospital to which the most severe cases were admitted. Less severe cases were admitted to makeshift wards in the so-called institute buildings, and the huts were used for convalescence. In his report, the Principal Medical Officer commented that this storm was likely to have exacerbated the impact of the outbreak and this is certainly plausible [3].

In addition to data limitations, the parameters used for the SEIR model also involve uncertainties; for example, we have no good data on the proportion of the young male population who were likely to be susceptible to this strain in 1918 (e.g. based on the possible residual immunity from the first wave of the pandemic or from previous influenza epidemics and pandemics). Also, the SEIR model involves a number of simplifying assumptions, including a single index case, homogeneous mixing, exponentially distributed residence times in infectious status categories, and isolation of the military camp.

### Estimating *R*_0_

The estimates for *R*_0 _in the range from 1.3 to 3.1 are the first such estimates for the 1918 pandemic outside Europe, the United States and Brazil, so far as we are aware. However, given the unique aspects of the military camp (crowded conditions and a young population with low immunity) it is quite likely that the *R*_0 _values estimated in our analysis might tend to over-estimate those for the general population. Nevertheless, this effect may have been partly offset by the camp policy of immediate hospitalisation upon symptoms, effectively reducing infective contacts.

Our estimated range for *R*_0 _is broadly consistent with estimates for this pandemic in the United States (a median *R*_0 _of 2.9 for 45 cities) [12]. Other comparable figures for the 1918 pandemic are: 1.7 to 2.0 for the first wave for British city-level mortality data [13]; 2.0, 1.6 and 1.7 for the first, second and third waves in the UK respectively [14]; 1.5 and 3.8 in the first and second waves in Geneva respectively [15]; and 2.7 for Sao Paulo in Brazil [16]. The upper end of our estimated range (*R*_0 _= 3.1) may reflect the differences between disease transmission in the general population (as per the above cited studies) and transmission in a crowded military camp with a predominance of young males.

Considered collectively, these *R*_0 _estimates for pandemic influenza in various countries are not particularly high when compared to the *R*_0 _estimates for various other infectious diseases [17]. This observation provides some reassurance that if a strain of influenza with similar virulence were to emerge, then there would be scope for successful control measures. Indeed, one model, using *R*_0 _values in the 1.1 to 2.4 range, has suggested the possibility of successful influenza pandemic control [18]. This was also the case for a model using *R*_0 _= 1.8 [19]. Nevertheless, at the upper end of the estimated range for *R*_0_, control measures may be more difficult, especially if public health authorities are slow to respond and they have insufficient access to antivirals and pandemic strain vaccines.

### The latent and infectious periods

The average latent and infectious periods were estimated to be in the range between 0.7 to 1.3 days, and 0.2 to 0.3 days, respectively. The infectious period is short compared to the period of peak virus shedding known to occur in the first 1 to 3 days of illness [20]. Other modelling work has used longer estimates, e.g. a mean of 4.1 days used by Longini et al. [18].

The fast onset and subsequent decline of the outbreak in the Featherston Military Camp, as compared to a national or city-wide outbreak, might possibly be due to relatively close habitation and a high level of mixing. The average time for infection between a primary and secondary case (the serial interval) is greatly shortened in this case. This could explain a short apparent infectious period, and a relatively large proportion of the serial interval in the latent state. Another possible explanation of the relatively short apparent infectious period for this outbreak is that it may reflect the limited transmission that occurred once symptomatic individuals were hospitalised on diagnosis – which was the policy taken in this military camp for all cases.

### The lag period from diagnosed illness to death

This analysis was able to estimate an approximate seven-day delay from reported symptomatic illness to the date of death at a population level. This result is suggestive that even in this relatively young population (largely of military recruits), an important cause of death was likely to have been from secondary bacterial pneumonia – as opposed to the primary influenza viral pneumonia or acute respiratory distress syndrome (for which death may have tended to occur more promptly). This finding is consistent with other evidence that a large proportion of deaths from the 1918 pandemic was attributable to bacterial respiratory infections [21]. This picture is also somewhat reassuring as it suggests that much of this mortality could be prevented (with antibiotics) if a novel strain with similar virulence emerged in the future.

## Conclusion

The *R*_0 _estimates in the 1.3 to 3.1 range are broadly consistent with others made for the 1918 influenza pandemic and are not particularly large relative to some other infectious diseases. This finding suggests that if a novel influenza strain of similar virulence emerged then it could potentially be controlled through the prompt use of major public health measures. These results also suggest that effective treatment of pneumonia could result in better outcomes (lower mortality) than was experienced in 1918.

## Competing interests

The author(s) declare that they have no competing interests.

## Authors' contributions

Three of authors were involved in initial work in identifying the data and analysing it from a historical and epidemiological perspective (PN, NW and MB). The other two authors worked on developing and running the mathematical model (GS, MR). GS did most of the drafting of the first draft of the manuscript with assistance from NW. All authors then contributed to further re-drafting of the manuscript and have given approval of the final version to be published.
